# Digital dance programs for Parkinson's disease: challenges and opportunities

**DOI:** 10.3389/fpsyg.2025.1496146

**Published:** 2025-01-23

**Authors:** Judith Bek, Deborah A. Jehu, Meg E. Morris, Madeleine E. Hackney

**Affiliations:** ^1^School of Psychology, University College Dublin, Dublin, Ireland; ^2^Centre for Motor Control, Faculty of Kinesiology and Physical Education, University of Toronto, Toronto, ON, Canada; ^3^Department of Community and Behavioral Health Sciences, Institute of Public and Preventative Health, School of Public Health, Augusta University, Augusta, GA, United States; ^4^Academic and Research Collaborative in Health and CERI, La Trobe University, Melbourne, VIC, Australia; ^5^Department of Medicine, Division of Geriatrics and Gerontology, Emory University School of Medicine, Atlanta, GA, United States; ^6^Atlanta VA Center for Visual and Neurocognitive Rehabilitation, Decatur, GA, United States; ^7^Birmingham/Atlanta VA Geriatric Research Education and Clinical Center, Decatur, GA, United States

**Keywords:** Parkinson's disease, digital healthcare, neurorehabilitation, telemedicine, dance

## Introduction

Dance provides therapeutic benefits for people with Parkinson's disease (PD) across motor and non-motor domains, including gait, mobility, mood, and cognition (McNeely et al., 2015; Shanahan, 2015; Bek et al., 2020; Carapellotti et al., 2020; Emmanouilidis et al., 2021). As a low-cost and widely accessible activity, dance can be a valuable adjunct to standard clinical treatment for PD. Digital provision of dance for PD has expanded significantly, accelerated by the COVID-19 pandemic (Bek et al., 2021a; Kelly and Leventhal, 2021; Morris et al., 2021, 2023). There is an ongoing need for accessible digital platforms for therapeutic activities like dance (Ellis and Earhart, 2021; Kelly and Leventhal, 2021) to provide for the growing PD population (Dorsey et al., 2018), including those in rural and remote communities without access to in-person programs. This article considers key challenges and potential solutions in digital dance for PD.

## Current evidence on the feasibility and outcomes of digital dance programs for PD

Preliminary evidence indicates that online dance can be safe and feasible for individuals with mild to moderate PD, showing good rates of attendance and adherence and no adverse events (Morris et al., 2021, 2023; Walton et al., 2022; Pinto et al., 2023; Delabary et al., 2024a). Advantages of the digital format noted by people with PD include the convenience of not traveling and the ability to practice more frequently (Bek et al., 2021a; Ghanai et al., 2021). Participants report enjoyment of digital classes (Morris et al., 2021; Walton et al., 2022) and a desire to continue with online dance alongside in-person classes (Bek et al., 2021a; Delabary et al., 2024b), as also noted for exercise classes (Bennett et al., 2023) and singing therapy (Tamplin et al., 2024) for PD.

Live online dance participation has been associated with improvements in functional mobility, anxiety, and depression (Shanahan et al., 2017; Walton et al., 2022; Pinto et al., 2023), affect (Ghanai et al., 2021), and quality of life (Walton et al., 2022) in people with PD. Participants with PD engaging in live and/or recorded digital dance programs during the pandemic self-reported multiple motor (e.g., balance, posture) and non-motor (e.g., mood, confidence) benefits (Bek et al., 2021a).

Digital formats thus show promise as a feasible and effective approach to dance for PD. However, the literature is limited, including small samples and different modes of delivery. As practice and research in this field continue expanding, it is important to consider how the digital environment might impact therapeutic benefits of dance programs. Dance is a multidimensional activity (Dhami et al., 2015; Christensen et al., 2017; Bek et al., 2022a) incorporating physical, cognitive, social, affective, and creative components that contribute to outcomes for people with PD. The following section outlines therapeutic elements of dance that differ between in-person and digital contexts, with possible solutions to address limitations and optimize the experience and outcomes of digital dance programs. Table 1 summarizes the key challenges and solutions.

**Table 1:** Therapeutic elements of dance that may be impacted in the digital environment.

**Elements of dance impacted by digital environment**	**Potential solutions**
*Social interaction:* reduced opportunities for communication with peers and instructors	Live online classes; post-class social time; small classes with regular attendees; breakout rooms for larger classes; forums to communicate outside of class
*Quality of instruction and feedback:* loss of one-to-one feedback and personalized adaptations; absence of volunteers or assistants to support	Live online classes; clear instructions with repetition; one-to-one or small group teaching; individual support from volunteers/assistants; time for questions and suggestions after class; use of AI for individualized monitoring and feedback
*Representation of movement and the body:* altered body perception caused by restricted space, observing oneself on screen, and absence of visual and tactile feedback from others and the environment	Instructions to focus on observation of instructor/other dancers to support observational learning; use of kinesthetic imagery to increase sensations of movement
*Music and rhythm:* reduced enjoyment and rhythmic cues without live music; reduced audio quality	Ensure appropriate volume and audio quality of teaching materials and home devices to optimize therapeutic effects of music; facilitate personalized music choices to increase enjoyment and engagement
*Aesthetics and creativity:* reduced opportunities for creative expression and escapism	Participants to contribute to choreographic themes; encourage the use of props at home to promote creativity; use of analogy/metaphorical imagery to enhance the aesthetic experience of dance and guide movement quality; use of AI to support selection of culturally relevant themes and music

## Therapeutic elements of dance that may be impacted in the digital environment

### Social interaction

The extent and nature of social interaction is altered by the absence of a group or partner in the digital environment (Bek et al., 2021a; Ghanai et al., 2021; Walton et al., 2022). Qualitative reports indicate that participants value the peer support, social comparison (Walton et al., 2022; Senter et al., 2024), and physical contact (Rocha et al., 2017; Delabary et al., 2024b) provided by in-person classes. Although enjoyment from social interactions can be compromised in digital programs (Emmanouilidis et al., 2021), meaningful social engagement can still be achieved. For example, virtual coffee time after class provides opportunities for discussion, questions, and feedback (Delabary et al., 2024b). Smaller live online classes with regular participants could also create a sense of community (Bek et al., 2022b). Social interaction in large classes could be supported by using “breakout” groups to facilitate discussion after class or by providing an online forum to promote interaction outside of classes.

### Quality of instruction and feedback

The effectiveness of instruction in the digital environment may be limited by factors including video/audio quality, viewing perspectives, and interaction with instructors (Bek et al., 2021a, 2022b; Delabary et al., 2024b; Tamplin et al., 2024). Feedback is important to ensure that movements are performed safely, to provide necessary adaptations, and to facilitate learning. A qualitative review of in-person dance for PD highlighted the value of the instructor-participant relationship (Senter et al., 2024). In contrast, reduced interaction with the instructor and the loss of one-to-one support were cited by participants with PD as disadvantages of digital classes (Bek et al., 2021a). These limitations may impact participants' motivation and confidence to engage as well as the potential for learning.

Quality instruction may be maintained in the digital environment through optimizing aspects of class design and production, such as slowing the teaching pace and repeating instructions (Delabary et al., 2024b). Live online classes are critical to enable participants to receive feedback. To facilitate high-quality feedback in digital programs, instructors could consider one-to-one or small group classes with volunteers or assistants to support participants during class. Participants should have opportunities to ask the instructor questions or make suggestions after class, which could be combined with social/coffee time (Bek et al., 2022a; Delabary et al., 2024b).

### Representation of movement and the body

Dancers frequently observe, imitate, mirror, and coordinate with others' movements (Blasing et al., 2012; Bek et al., 2020). These processes engage the brain's motor system to facilitate movement and learning (Hardwick et al., 2018; Chye et al., 2022). Interventions based on action observation and motor imagery have shown positive effects in PD (Caligiore et al., 2017; Bek et al., 2021b; Mezzarobba et al., 2024), and qualitative data suggest that observation and imagery may be effectively implemented within dance for PD (Bek et al., 2022a). Additionally, awareness of the body is an important element of dance, and in-person dance training may enhance body awareness in PD (Hadley et al., 2020). Body perception may be altered in the digital space (Delabary et al., 2024a), for example through seeing oneself on screen or having a more restricted area within which to move.

Self-report data indicates that many people with PD can engage in observational learning and use imagery to enhance the outcomes of digital dance participation (Bek et al., 2021a, 2022b). These processes could be supported in digital programs through specific instructions to increase attention to the movements of the instructor and other dancers and by encouraging participants to imagine the sensations of the demonstrated movements (i.e., kinesthetic imagery).

### Music and rhythm

Music and rhythm are integral to most forms of dance and may contribute to beneficial effects for people with PD. Rhythmic cueing can support gait in people with PD (de Dreu et al., 2011; Nombela et al., 2013). Music promotes dopamine release in the basal ganglia (Salimpoor et al., 2011), and the beneficial effects of music in PD extend beyond rhythm to influence affect and motivation (Karageorghis et al., 2020; Tamplin et al., 2020, 2024). Music can also evoke motor imagery in people with PD (Poliakoff et al., 2023), and participants could potentially utilize the music from dance classes as an internalized cue to support movement in daily life (Bek et al., 2022a; Jola et al., 2022). People with PD enjoy the music accompanying dance classes and have expressed a preference for live music (Ghanai et al., 2021), which participants miss when dancing at home (Bek et al., 2021a).

In a recent study examining the feasibility of a one-on-one digital dance program for PD, participants worked with the instructor to select music for classes (Morris et al., 2021). Although it may be difficult to effectively tailor music to preferences in a group online class, different choices could be accommodated across a series of classes. Instructors should ensure appropriate music quality and volume (Delabary et al., 2024b), and participants could be supported to optimize the audio settings of their devices.

### Aesthetics and creativity

The creative aspects of dance differentiate it from other forms of physical activity (Rocha et al., 2017; Fontanesi and DeSouza, 2021; Bek et al., 2022a). Dance programs for PD often feature communicative expressions and gestures, storytelling, and props. Qualitative data indicate that participants enjoy the creativity and escapism offered by dance (Bek et al., 2022a; Walton et al., 2022) and that artistic aspects of dance are diminished in the digital environment (Walton et al., 2022). Additionally, skin conductance measures have indicated that dance may increase physiological arousal compared to an exercise program of similar aerobic intensity (Fontanesi and DeSouza, 2021), suggesting an emotional response to the artistic experience of dance. To enhance the aesthetic and creative dimensions of digital dance programs, participants could be encouraged to contribute to choreographic themes and stories or use props during home practice. Instruction could also incorporate analogy and metaphorical imagery, which has been associated with positive outcomes of digital dance participation (Bek et al., 2021a).

## Future directions for research and development

Further to the therapeutic elements of dance discussed above, there are important practical considerations in designing digital programs. Ensuring safety in the digital environment is critical, particularly considering gait and balance difficulties in PD, which can increase fall risk (Camicioli et al., 2023). People with PD who attend dance classes are at different stages of disease progression and can have different needs. They may also have different infrastructures at home to support online physical activity. It is important for health professionals and instructors to ensure that participants are safe to engage in home-based dance training, particularly those with greater disease severity who experience postural instability. Checklists have been devised to assist this process (see Morris et al., 2021, 2023). It is also advisable to use a checklist before each session to assess safety of the home environment and note procedures for dealing with adverse events.

Technical barriers relating to the hardware, software, or connectivity required for online classes must also be considered for both participants and instructors (Bek et al., 2021a, 2022b; Walton et al., 2022; Delabary et al., 2024b). To increase the accessibility of dance programs, alternative options could be offered to accommodate different abilities and preferences. While most participants may prefer live online classes that provide greater social interaction, others prefer recorded videos that offer flexibility and enable self-paced or repeated practice, or appreciate having both live and recorded options (Bek et al., 2021a). Pre-recorded DVDs or dance instruction by telephone^1^ could provide valuable resources for individuals in rural communities without reliable internet access or a suitable electronic device, and these should also incorporate appropriate safety checks. A choice of different digital platforms for accessing classes could also increase participation by providing options that participants are familiar with (Delabary et al., 2024b). Participants could also be supported with training or guidance to use the required technology before joining a program.

Involving people with PD in the co-design and development of programs increases the relevance and could enhance outcomes of therapeutic activities like dance (Quinn et al., 2010; Morris et al., 2021; Bek et al., 2022a). Participant input is particularly valuable in digital programming to understand specific preferences, needs, and challenges. High levels of attendance and enjoyment of co-designed online dance classes have been reported (Morris et al., 2021), and patient input can enhance the accessibility and usability of digital technology platforms for home-based training (Bek et al., 2021b).

Finally, future research and development should capitalize on the opportunities offered by artificial intelligence (AI) to enhance and personalize digital therapies (Amjad et al., 2023), including the possibility to provide participants, dance instructors, and healthcare professionals with performance data and feedback. For example, computer vision and machine learning techniques could be used to measure changes or improvements in movement and adjust programs to align with individuals' ability levels. Participants could also receive individualized encouragement and guidance. AI could also allow instructors to tailor classes to different languages, cultures, and geographical locations, for example through translating instructions or suggesting culturally relevant music and themes.

## Conclusions

Current evidence indicates that digital dance is feasible and enjoyable for many people with PD, and preliminary findings suggest that positive outcomes can be achieved in the online environment. Digital platforms can increase the reach of dance programs, although barriers to access remain. However, the evidence so far is limited to small-scale studies and self-report data, and the outcomes of digital and in-person programs have not been directly compared. Further research is needed to understand the impact of digital dance across domains and at different disease stages, including longer-term outcomes. In the meantime, this article outlines possible solutions to help maintain therapeutic benefits of dance in the digital environment.

## Footnotes

^1^These options are already offered by Dance for PD^®^ https://danceforparkinsons.org/.
